# Implementation and evaluation of team science training for interdisciplinary teams in an engineering design program

**DOI:** 10.1017/cts.2021.788

**Published:** 2021-05-14

**Authors:** Erin Abu-Rish Blakeney, Soyoung Kang, Katrina Henrikson, Jonathan T. C. Liu, Eric J. Seibel, Jennifer Sprecher, Nicole Summerside, Mia T. Vogel, Brenda K. Zierler, Jonathan D. Posner

**Affiliations:** 1 School of Nursing, University of Washington, Seattle, WA, USA; 2 Institute of Translational Health Sciences, University of Washington, Seattle, WA, USA; 3 Mechanical Engineering, University of Washington, Seattle, WA, USA; 4 Bioengineering, University of Washington, Seattle, WA, USA; 5 Laboratory Medicine and Pathology, University of Washington Medical Center, Seattle, WA, USA; 6 Electrical and Computer Engineering, University of Washington, Seattle, WA, USA; 7 Oral Health Sciences, University of Washington, Seattle, WA, USA; 8 Brown School, Washington University, St. Louis, MO, USA; 9 Institute for Clinical and Translational Sciences, Washington University, St. Louis, MO, USA; 10 Center for Public Health Systems Science, Washington University, St. Louis, MO, USA; 11 Chemical Engineering, University of Washington, Seattle, WA, USA; 12 Family Medicine, University of Washington, Seattle, WA, USA

**Keywords:** Team science, engineering, innovation, education, translational workforce

## Abstract

**Introduction:**

Interdisciplinary academic teams perform better when competent in teamwork; however, there is a lack of best practices of how to introduce and facilitate the development of effective learning and functioning within these teams in academic environments.

**Methods:**

To close this gap, we tailored, implemented, and evaluated team science training in the year-long Engineering Innovation in Health (EIH) program at the University of Washington (UW), a project-based course in which engineering students across several disciplines partner with health professionals to develop technical solutions to clinical and translational health challenges. EIH faculty from the UW College of Engineering and the Institute of Translational Health Sciences’ (ITHS) Team Science Core codeveloped and delivered team science training sessions and evaluated their impact with biannual surveys. A student cohort was surveyed prior to the implementation of the team science trainings, which served as a baseline.

**Results:**

Survey responses were compared within and between both cohorts (approximately 55 students each Fall Quarter and 30 students each Spring Quarter). Statistically significant improvements in measures of self-efficacy and interpersonal team climate (i.e., psychological safety) were observed within and between teams.

**Conclusions:**

Tailored team science training provided to student-professional teams resulted in measurable improvements in self-efficacy and interpersonal climate both of which are crucial for teamwork and intellectual risk taking. Future research is needed to determine long-term impacts of course participation on individual and team outcomes (e.g., patents, start-ups). Additionally, adaptability of this model to clinical and translational research teams in alternate formats and settings should be tested.

## Introduction

There is a strong foundation of theory and data demonstrating the need for interdisciplinary teams to be competent in the principles of teamwork [1,2]. Interdisciplinary teams that have alignment and a shared understanding of goals, processes, and roles and strong interpersonal skills and relationships have been found to be more productive and generate research that is more innovative and impactful (i.e., highly cited) compared to research published by authors within a single group or discipline [1,3–5]. At the same time, challenges encountered by interdisciplinary teams have been well documented and include the lack of a common vocabulary, poor communication, and misaligned coordination resulting in duplicated efforts, delays, and frustration [1].

The need for effective teams and team training has long been a focus in business, military, and industry settings and is an increasing focus in translational research and healthcare [1]. It is widely accepted that the challenges faced by teams across contexts are similar and thus that interventions to improve teamwork are likely generalizable to a variety of settings [6–8]. Meta-analyses of team training interventions also conclude that team training can improve team effectiveness [7,8]. However, there remains great heterogeneity in teamwork and collaboration terms, measurements, and frameworks that make it difficult to synthesize the evidence in this area. There is consequently a lack of best practices of how to introduce and facilitate the development of effective functioning within interdisciplinary teams, including newly formed teams and teams of students and health professionals (which we hereafter refer to as student-professional teams) in educational settings [9]. To address these gaps, we codeveloped, implemented, and evaluated a context-specific team science curriculum for student-professional teams in an existing year-long project-based Engineering Innovation in Health (EIH) program at the University of Washington (UW). The purpose of this manuscript is to report on our efforts to foster individual and interpersonal team learning and thereby enhance effectiveness among new EIH teams with the overarching goals of accelerating clinical and translational research and improving health. This approach is consistent with group process team learning models articulated by Edmondson *et al.* wherein team learning and performance both influence and are influenced by the individuals within a team and that team’s interpersonal climate [10,11].

## Background

The UW Institute of Translational Health Sciences (ITHS) Team Science Core has the goal of carrying out research and training in team science [12]. Team science is defined as “scientific collaboration (i.e., research conducted by more than one individual in an interdependent fashion), including research conducted by small teams and larger groups” [1] and “a collaborative effort to address a scientific challenge that leverages the strengths and expertise of professionals trained in different fields” [13]. The ITHS Team Science Core is composed of educators and researchers with expertise in team science, interprofessional education, lean project management, faculty development, collaborative practice, leadership, nursing, health services research, and engineering. This ITHS Core is working with 3 interdisciplinary groups in diverse clinical and translational settings (e.g., education, clinical, and research) to develop and evaluate team science training. One of the education groups this Core is working with is UW’s EIH program.

EIH is a highly-rated three-quarter longitudinal academic program in the College of Engineering at the UW that promotes interdisciplinary collaboration between engineering and the health sciences (https://eih.uw.edu). The focus of the EIH program is to develop innovative technical solutions to pressing clinical and translational health challenges. Undergraduate and graduate students across engineering disciplines (e.g., mechanical, electrical, human-centered design, bio, chemical, and materials science) are partnered with health professionals (e.g., physicians, nurses, dentists, pharmacists) to solve unmet health challenges. Unmet health challenges are proposed each year by health professionals and, if selected, health professionals agree to spend 1–3 hours/week working on the project with student teams. The program follows a need-based design philosophy that begins with an unmet health need and examines the stakeholders, market opportunity, intellectual property, FDA regulations, and reimbursement [14]. Teams of 3–5 students work closely with the health professionals who proposed the topic to develop a deep understanding of the unmet health need in the first quarter and then develop a functional prototype, intellectual property, and an early-stage business plan in the second and third quarters of the academic year. Prior to the collaboration between the EIH program and the ITHS Team Science Core, teams received guidance on improving functioning on a case-by-case basis, and often after challenges had arisen. Each year of the program, while the majority of teams were successful in achieving course goals, EIH faculty observed some teams struggle without a clear difference in team composition or project promise. Further, EIH faculty observed that teams that appeared to be high functioning had better outcomes than those that appeared to function poorly (e.g., projects continuing from Fall to Winter/Spring and beyond the course, quality of innovation, likelihood of measurable outputs). For these reasons, the EIH faculty embraced the opportunity to partner with ITHS to test and evaluate the integration of purposeful team science training into the curriculum to strengthen an already strong translational program using a collaborative, appreciative inquiry approach [15].

## Methods

### Nomenclature

The EIH program is comprised of sequential courses across the academic Fall, Winter, and Spring quarters, which we hereafter refer to as Q1, Q2, and Q3 for the respective quarters. We refer to the first year of the study as Year 0 (Y0) or the baseline year. During the baseline year, observations and surveys took place but there was no team science training implemented in the course. Team science training was incorporated during the second year of the study, which we refer to as Year 1 (Y1) or the team science training year.

### Baseline Data Collection and Needs Assessment

In the baseline year, we conducted classroom observations and developed and administered surveys to the students about their experiences working in past teams as well as with their current EIH project teams. Observers were members of the Team Science Core and utilized a semi-structured observation tool to gather information about the course content and approach. In addition, observers noted areas where team science concepts were already present and could be amplified or where they were not clearly present and could be beneficial to EIH teams over the course of their projects and the academic year. Survey questions were adapted from existing translational team competency domains and previous surveys developed by the Team Science Core and the National Cancer Institute’s (NCI) Team Science Toolkit Tool Library [16–22]. Specifically, from the NCI Team Science Toolkit, question stems from the Research Collaboration Survey and the Interpersonal Collaboration Survey were tailored to this study after course observations [17]. A focus of our efforts was to identify questions that could be used to gather information about student’s self-efficacy for working in teams and also to assess interpersonal team climate (i.e., psychological safety) over the course of the academic year. Self-efficacy is defined by “people’s beliefs about their capabilities to produce effects,” and self-efficacy to work in teams is an important aspect of teamwork [6,23]. Team climate questions focused on aspects of interpersonal collaboration that align with the concept of psychological safety, including trust and information exchange [11,24]. Face validity for questions was assessed through consultation with experts in evaluation and team science. Content validity was tested by EIH faculty, teaching assistants and members of the ITHS Team Science Core. Minor adjustments were made to the questions to be suitable for the EIH course and the student population [25]. Final survey questions were a mix of Likert-type and short answer and included questions about student sociodemographic characteristics (see Appendix A for the surveys).

Observations and surveys from the baseline year (Y0) confirmed course evaluation data that indicated that the course was highly interactive and well-liked by the majority of students, as well as revealed opportunities for integration of team science content in specific areas of the EIH curriculum. Baseline data and a proposed list of team science topics and approaches was shared with EIH faculty, who also participated in the annual ITHS Team Science Boot Camp training. EIH faculty and members of the Team Science Core discussed the baseline survey results and potential topics and approaches and agreed upon a plan for integrating team science content and approaches for the intervention year (Y1). Topics included: building trust and mutual respect among team members, effective communication, time/project management, conflict resolution, and recognizing the diverse strengths of team members. Given the focus in the team science literature on the importance of shared team processes and the value of reflection, an overarching theme of the team science intervention was to create opportunities for planning, action, and reflection as a team [1,24,26].

After the baseline year, members of the Team Science Core delivered the team science training content, while EIH faculty observed. This approach, in addition to attending the Team Science Boot Camp training, prepared EIH faculty to deliver team science training in subsequent years. The student training approach was highly interactive with short (15-minute or less) didactic content followed by facilitated exercises (application of content). This structure was informed by experiential learning and social learning theories [27,28]. The freely available library of Liberating Structures [29] was used to plan the interactive portions of team science sessions (see Appendix B for session and tool details). The timing of team science training sessions was planned to support both individual and team learning at key points in the year.

**Fig. 1.**
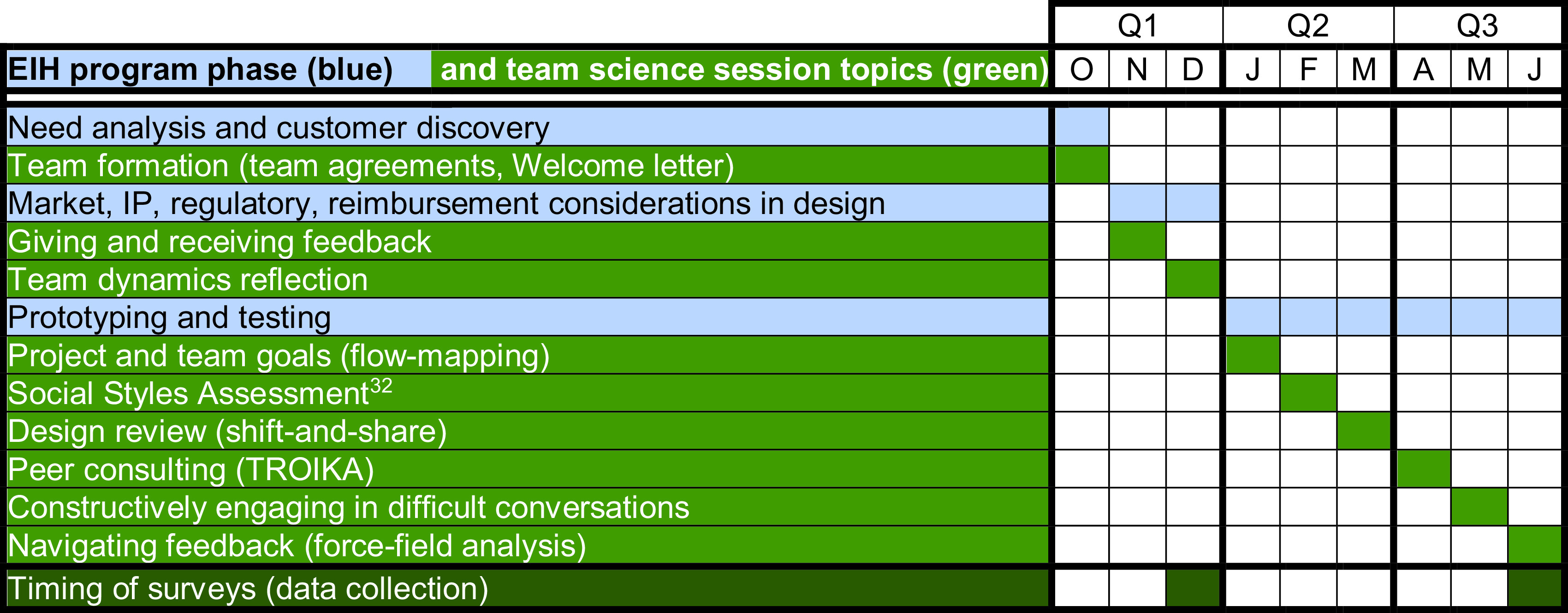
A timeline of team science training.

The evaluation surveys were administered to students at the end of Q1 and Q3 for both baseline and team science training years (Fig. 1). The Q1 survey asked students to respond to questions about their previous experiences working in teams and experiences working with their EIH project team during Q1. In contrast, the Q3 survey asked students to respond to questions about their experiences working with the EIH project team through the remainder of the 3-quarter program.

### Delivery of Team Science Training Sessions

The team science training sessions were integrated with the EIH curriculum throughout Y1. For example, early in Q1, as the EIH students were building a critical foundation in engineering design through need analysis and customer discovery, the team science session for team formation was delivered in class as part of a lecture. Example activities for team formation included developing team agreements [30] and drafting a welcome letter [31] for the student-professional teams to establish expectations and map out goals for the project. As the course progressed through Q1, students learned about and explored various external design considerations (e.g., market, intellectual property, regulatory, and reimbursements). Midway through Q1, a team science training session on ‘Giving and Receiving Feedback’ was delivered in class just prior to midterm presentations in which teams presented their findings to their peers. Three team science sessions were delivered in the class each quarter. More information regarding each session can be found in Appendix B. Figure 1 shows a timeline of when the team science sessions were implemented and delivered throughout the EIH program. As noted previously, team science sessions were facilitated by members of the ITHS Team Science Core.

### Assessment and Evaluation of Teams and Team Science Sessions

We administered the Q1 survey at the end of the quarter. Students were asked to think about their team experience prior to EIH and rate how capable they were to “speak up in team meetings,” ‘advocate for multiple points of view,’ “resolve conflicts with peers and other collaborators,” and other statements related to self-efficacy (the full list of statements is provided in Appendix A). The surveys used a 5-point Likert-type scale from “not at all capable” to “very capable.” Students were also asked to respond to the same questions in regards to their team experience after participation in EIH and working with their project team. We also asked students to rate their level of agreement with statements related to the interpersonal climate of their EIH project team. Q3 surveys (administered at the end of Q3) again asked students about their capability to work effectively in their project teams and about their team’s interpersonal climate (i.e., psychological safety).

The surveys were administered online via REDCap [32,33]. Those who did not respond or who indicated that a component of the questionnaire was “not applicable” were excluded from analysis of that particular section. Because survey responses were anonymous, linking individual responses between periods (e.g., Y0, Q1 and Q3) was not possible. Additionally, the student groups between Y0 and Y1 were independent as students only take the course one time.

### Data Analysis

We used descriptive statistics to summarize student demographics and survey responses within and between years. We did not conduct a power analysis beforehand as the total class population was included in the study. Median and interquartile range (IQR) of responses to Likert-type questions were calculated, in accordance with appropriate approaches for analysis of this type of skewed Likert-type response data [34–38]. We reported median as the measure of central tendency for these analyses as opposed to the mean which is sensitive to outliers. Survey results for Likert-type questions were compared between quarters (e.g., Y0, Q1 vs Q3) to show the changes in individual self-efficacy and interpersonal team climate throughout the year. In addition, survey results were compared between years (e.g., Y0 vs Y1) to determine if there were differences in team functioning between baseline and team science training years. Mann-Whitney U Tests (independent samples of Wilcoxon Rank Sum Tests) were used to test these comparisons.

### Human Subjects

All study activity was carried out in accordance with approved ethical guidelines and was deemed exempt from the Institutional Review Board and Human Subjects Division at the UW.

## Results

### Student Demographics across Years

The demographics of students who responded to the surveys are provided in Table 1 for Y0 (baseline year) and Y1 (team science training year). Note that the number of students varies each program year and from Q1 to Q3 of each program year (not all students continue in the program after the fall introductory quarter). However, in all quarters, response rates were very high (96%–100%). In both years, approximately two-thirds of students identified as male. A slightly higher proportion of male-identifying students continued in the program for the entire year relative to students who identified as female. In Y1, a higher proportion of students were undergraduate students than in Y0. In the Q1 surveys, students were asked to select the types of settings in which they had worked on group projects prior to participation in EIH. This data provides insight into the context from which students entered the EIH program. Despite the differences in demographics in the student composition between Y0 and Y1, Table 1 shows a relatively comparable level of prior group project experience across the years, with over 90% of students reporting prior group experience in engineering at the college level. The high proportion of students reporting previous experience working on teams was expected because all of the students were either enrolled in a graduate engineering program or in their fourth year of engineering education. All students reported some experience working in groups or teams. Table 1 summarizes results from cohorts across the years and suggest a high degree of similarity regarding the student’s team experiences between the cohort years.

**Table 1.** Gender, education level, and prior group project experience of the EIH students

	Y0 (baseline)		Y1 (team science training)	
	Q1 (N = 57) (%)	Q3 (N = 35) (%)	Q1 (N = 53) (%)	Q3 (N = 30) (%)
Response rate	100	100	96	100
Gender				
Male	63	66	62	67
Female	32	23	30	27
Prefer not to say	5	11	2	7
Level				
Undergraduate	47	54	70	77
Graduate	47	40	26	17
Prior group project experience				
High school classes	72		75	
College (≠ eng.)	77		75	
College (= eng.)	96		94	
Work or volunteer	63		68	
N/A	0		0	

Prior group project experience was only asked in the Q1 survey.

Note that at the end of Q1, projects and students are down-selected to roughly half for each, thus the class size, N, is smaller in Q3.

### Individual Self-Efficacy Working with Project Teams

Given that all students had group project experience prior to joining EIH (Table 1), we asked students to rate their self-efficacy working on project teams and evaluated the differences before, during, and after participation in EIH. This assessment provides insight into improvements in self-efficacy each year. Table 2 shows median and IQR for each of the eight self-efficacy statements. Self-efficacy was rated at a median of four or five by most students at all time points with improvements from the beginning to the end of the year during both years. Specifically, during the baseline year, the number of statements having a median of five increased from two to four, whereas during Y1 the number of statements having a median of five increased from one to eight.

**Table 2.** Median and interquartile range (IQR) of responses to self-efficacy statements during Y0 (left column block) and Y1 (middle column block) and comparisons within and between years using Mann–Whitney U tests (shaded columns)

	**Y0 (baseline)** **Q1 (N = 57)** **Q3 (N = 35)**			**Y1 (team science training)** **Q1 (N = 53)** **Q2 (N = 30)**			Testing for differences between years	
	Q1 before, median (IQR)	Q3 after, median (IQR)	Q1 before & Q3 after	Q1 before, median (IQR)	Q3 after, median (IQR)	Q1 before & Q3 after	Q1 before (Y0 & Y1)	Q3 after (Y0 & Y1)
1. Clarify language differences across disciplines/back-grounds	4 (1)	5 (1)	P = 0.02	4 (2)	5 (1)	P = 0.04		
2. Collaborate with team members with different working styles	4 (1)	4 (1)		4 (1)	5 (1)	P = 0.01		P = 0.04
3. Have your voice heard in team meetings	4 (1)	5 (1)		4 (1)	5 (0)	P =< 0.001		P = 0.02
4. Advocate for multiple points of view	4 (1)	4 (1)		4 (1)	5 (1)	P = 0.03		P = 0.02
5. Resolve conflicts with peers and other project collaborators	4 (1)	4 (1)		4 (2)	5 (1)	P = 0.04		
6. Recognize team members’ strengths	4 (1)	4 (1)		4 (1)	5 (1)	P = 0.04		
7. Effectively contribute in team meetings	5 (1)	5 (1)		4 (1)	5 (1)	P = 0.04		
8. Speak up in team meetings	5 (1)	5 (1)		5 (1)	5 (0)	P =< 0.001		

Responses are based on students reporting their level of self-efficacy on a 5-point Likert-type scale from “not at all capable” (1) to “very capable” (5).

P-values are derived from independent samples Mann–Whitney U Tests; P-values are only shown for statistically significant differences.

### Within Years Comparison

During Y0, from Q1 to Q3, improvements were statistically significant using a Mann−Whitney *U* test for only one self-efficacy statement about capability to “clarify language differences across disciplines/backgrounds” (Q1 median = 4, Q3 median = 5; z = −2.34; P = 0.02). In contrast, during Y1, there were statistically significant improvements from Q1 to Q3 for all eight self-efficacy statements following team science training (Table 2).

### Between Years Comparison

**Fig. 2.**
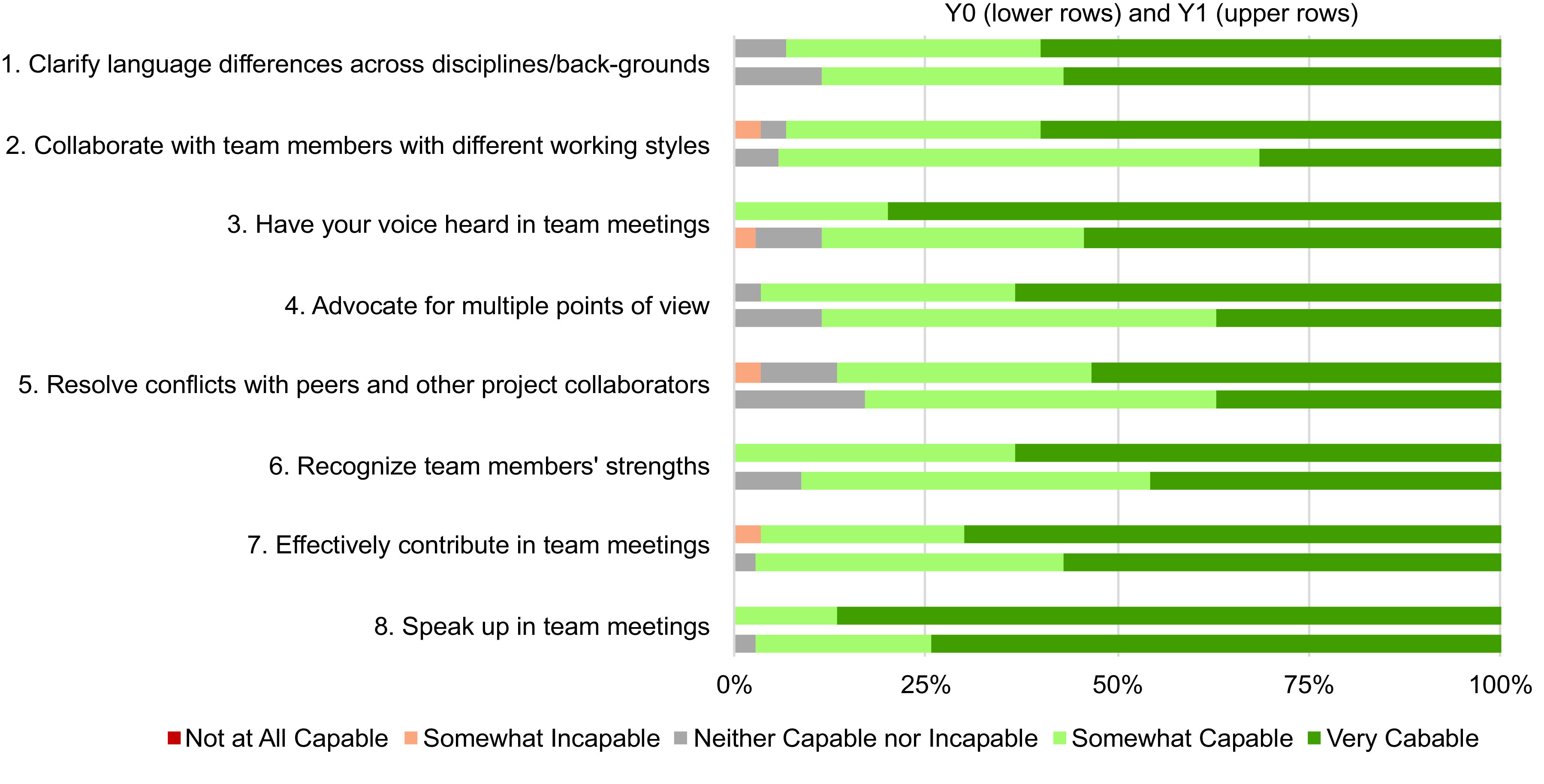
A visual comparison of responses to statements related to self-efficacy between Q3 for Y0 (lower rows) and Y1 (upper rows). Responses are based on students reporting their level of capability on a 5-point Likert-type scale.

When comparing “Q1 before” medians between Y0 and Y1, there were no significant differences, suggesting that these groups were similar prior to participation in the EIH program. When comparing Q3 responses between Y0 and Y1, statistically significant improvements were identified for three self-efficacy statements: “advocate for multiple points of view, ‘having your voice heard in team meetings,’ and collaborating with team members who have different working styles.” A visual comparison of responses to statements related to self-efficacy illustrates higher rates of students responding “very capable” for all statements in Y1 compared to Y0 at the time of the Q3 surveys (Fig. 2).

### Interpersonal Team Climate

Students were asked to rate their level of agreement with ten statements related to interpersonal team climate (i.e., psychological safety) at the end of Q1 (after they had worked with their EIH team for the first quarter) and at the end of Q3 (after they had worked with an EIH team for three quarters). Table 3 shows median and IQR for agreement with each statement.

**Table 3.** Summary of responses – team climate statements (median and interquartile range (IQR)) during Y0 (left column block) and Y1 (middle column block) and comparisons within and between years using Mann–Whitney U tests (shaded columns)

	**Y0 (baseline)** **Q1 (N = 57)** **Q3 (N = 35)**			**Y1 (team science training)** **Q1 (N = 53)** **Q3 (N = 30)**			Testing for differences between years	
	Q1 median (IQR)	Q3 median (IQR)	Q1 & Q3	Q1 median (IQR)	Q3 median (IQR)	Q1 to Q3	Q1 (Y0 & Y1)	Q3 (Y0 & Y1)
1. Communication with my team members outside of class was easy	4 (2)	4 (2)		4.5 (1.5)	5 (1)		P = 0.01	
2. Our project team has a climate of collaboration and trust	4 (2)	4 (1)		5 (1.5)	4.5 (1.0)		P = 0.04	P = 0.02
3. I had a desire to know my teammates on a personal level	4 (2)	4 (2)		4 (2)	4 (1)		P = 0.03	
4. Having a successful project was a priority for me	5 (1)	5 (1)		5 (1)	4.5 (1)			
5. I felt comfortable giving my team members feedback	4 (1)	4 (1)		5 (1)	4 (1)			
6. I was comfortable showing gaps in my knowledge with my team	4 (1)	4(1)		5 (1)	5 (1)			
7. Our project team has been successful working together	4 (1)	4 (1)		5 (1)	4 (1)			
8. I felt comfortable receiving feedback from my team members	5 (1)	5 (1)		5 (1)	5 (1)			
9. Building effective relationships with my team members was a priority for me	4 (1)	4 (1)		4 (1)	4 (1)			
10. Team members on my project had a high level of mutual trust	4 (2)	4 (1)		4 (2)	4 (1)			

Responses are based on students reporting their level of agreement on a 5-point Likert-type scale from “strongly disagree” (1) to “strongly agree” (5).

### Within Years Comparison

Similar to the results for self-efficacy (Table 2), median agreement scores were relatively high with a median of four or five for all statements at each time point within each year. There were no significantly different changes in agreement about team climate from Q1 to Q3 during either year.

### Between Years Comparison

**Fig. 3.**
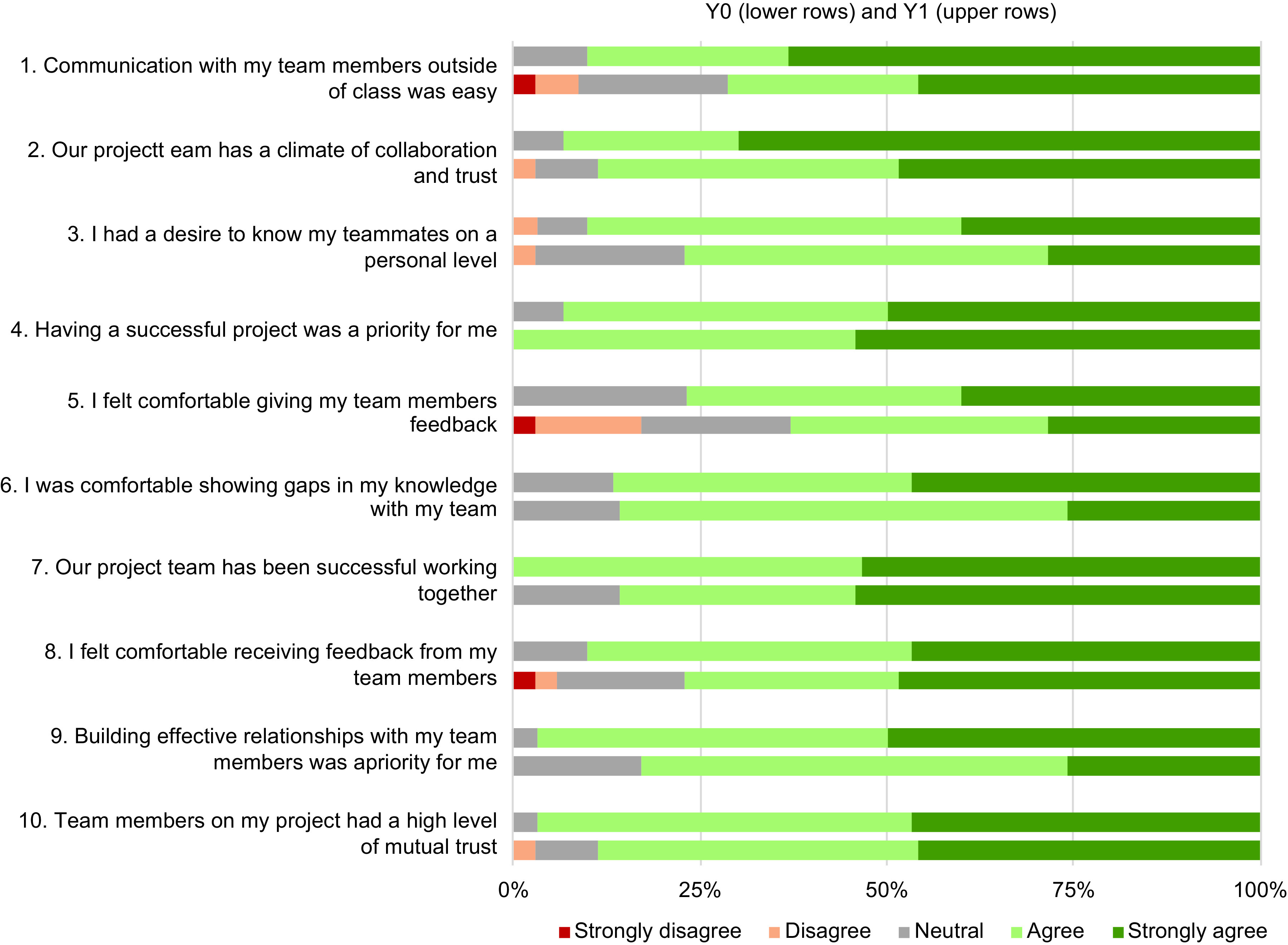
A visual comparison of Q3 responses to statements related to interpersonal team climate between Y0 (lower rows) and Y1 (upper rows). Responses are based on students reporting their level of agreement on a 5-point Likert-type scale.

When comparing Q1 between Y0 and Y1, there were statistically significant improvements in agreement with three statements: “our project team has a climate of collaboration and trust, ‘I had a desire to know my teammates on a personal level,’ and communication with my team members outside of class was easy.” When comparing Q3 between Y0 and Y1, there was only one statement for which there were statistically significant improvements “our project has a climate of collaboration and trust” (Y0/Q3 median = 4, Y1/Q3 median= 4.5, z = −2.03, P = 0.04). A visual comparison of responses reveals a shift toward higher levels of agreement (and less disagreement) with these statements at the end of (Q3) Y1 compared to Y0 (Fig. 3). For example, in Fig. 3, for statement 2 “Our team has a climate of collaboration and trust” saw a decrease from 2.9% to 0.00% in the proportion of respondents who disagreed with this statement and an increase of 21% in the proportion of respondents who indicated “strongly agree” in Y1 compared to Y0. In each case, except for question 4 “having a successful project was a priority for me,” the proportion of respondents who indicated agree or strongly agree increased and the proportion that indicated neutral, disagree, or strongly disagree decreased (in several cases to zero).

### Utility of Team Science Sessions

**Fig. 4.**
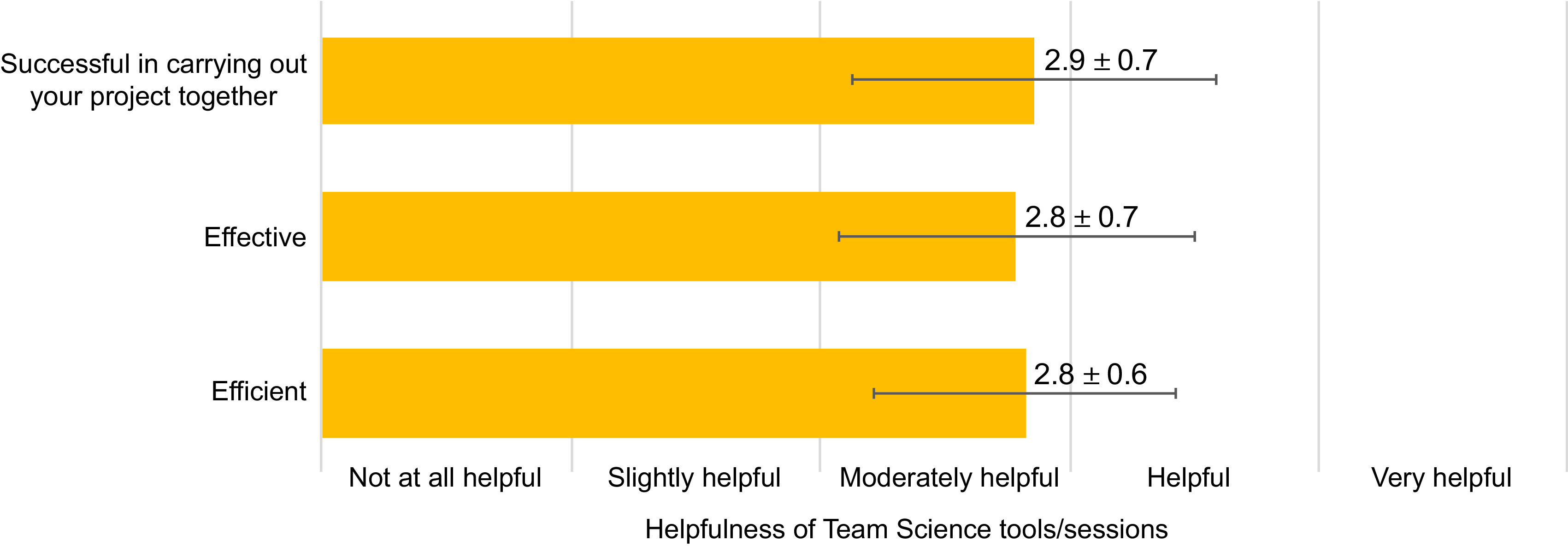
Helpfulness of team science sessions for team efficiency, effectiveness, and success as reported by students at the end of Y1. Responses are based on student reporting level of session helpfulness on a 5-point Likert-type scale.

After completing the EIH program with the team science training sessions in Y1, students were asked to indicate the extent to which the team science sessions assisted the project team to become more efficient (i.e., well-organized, minimum wasted effort, reaching milestones in a timely manner); effective (i.e., successful in producing desired or intended result, completing milestones); and successful in carrying out the project together. Figure 4 shows the mean of the student responses on a 5-point Likert-type scale from “not at all helpful” (1) to “very helpful” (5) for evaluating the helpfulness of team science sessions and shows that overall students found the sessions to be helpful.

## Discussion and Summary

Baseline data from Y0 observations and surveys provided an opportunity to study EIH teams without any intentional team science content. We observed from the baseline data that even without intentional team training, improvements in self-efficacy were made over the course of the year-long program, showing that many participants in this popular program build their teaming skills with or without team science training. Improvements in the student’s self-efficacy were noticeably enhanced with team science training as demonstrated by statistically significant improvements from Q1 to Q3 for all eight of the self-efficacy questions during the team science training year. We also identified improvements in several team climate questions that focused on interpersonal aspects of the teams. In particular, when comparing the baseline and team science training years, we identified statistically significant increases at the end of both Q1 and Q3 for the question “Our team has a climate of collaboration and trust.” This question aligns with the concept of psychological safety as described by Edmondson *et al.* [10,11,24].

Changes to answers on the negative end of the Likert-type scales for both self-efficacy and team climate were also observed and are an intriguing area of future study. For example, in Fig. 3, we see substantial changes in statements related to team communication and giving and receiving feedback. In the baseline year, 3% strongly disagreed, 14% disagreed, and 20% were neutral (total 37%) in response to the statement “I felt comfortable giving my team members feedback.” In contrast, in Y1 no respondents disagreed or strongly disagreed with this statement and only 23% (total 23%) responded that they were neutral. This suggests that the team science training content and opportunities to practice giving and receiving feedback helped lead to these results. Looking ahead in team science training, explicitly tracking changes in “negatives” that are known to challenge effective teamwork will likely be an important approach.

We noted instances in which improvements from team science training were not as substantial as we expected. One likely explanation for this, known as the Kruger-Dunning effect [39], is that students in the baseline year may have responded more confidently to survey questions when they did not know the team science principles/material, whereas those who were introduced to the team science content may have responded with more moderate confidence in themselves as they became both more skilled and more self-aware. These findings are consistent with other literature about training effects [39] and suggest that, following team science training, students may have more informed views of their own and their team’s effectiveness and more realistic expectations about next steps for their projects. For example, following Y1, fewer students indicated that they planned to continue working together in a team than in previous years, but a higher proportion of those teams did continue working together (see Appendix C for expected project outcomes). This presents challenges to objectively comparing survey responses between the baseline and team science training years and future studies would be strengthened by addressing these measurement challenges (i.e., with a control group and/or in the use of mixed methods).

In addition to the survey-based evaluations that we carried out in both the baseline and team science training years, we have identified several areas in which teams appear to be on track to demonstrate measurable improvements in program outcomes such as number of provisional patents submitted, start-up companies formed, teams continuing to work together, and funds raised (see Appendix C). For example, in our preliminary analyses, we have found that since 2013, the year in which the EIH program was founded, on average one team out of eight each year submitted a patent application, formed a startup, or carried projects through to clinical applications. In contrast, in the year after implementing the team training, four teams have submitted patent applications, are in the process of forming a startup, or are now working closely with health care professionals and device experts to evaluate devices for clinical use. These promising preliminary reports should be further studied in a longitudinal manner. Changes may carry increased significance since the team science trainings resulted in less in-class time for the teams to conduct traditional design, prototyping, and engineering analysis, which was typically incorporated into the team work time following delivery of lecture.

We acknowledge limitations of both our data and methods that influence rigor and generalizability. This effort was carried out in a real-world educational setting where it was not practical to identify concurrent control and intervention groups within the same EIH cohort. To mitigate this, we collected data for a year prior to the introduction of team science content and have used that data as a comparator for the intervention year. Demographic data demonstrate similarities between the two cohorts (Table 1), and minimal changes to the engineering content increase the likelihood that the changes that we identified were due to the implementation of team science training. Anecdotal feedback from EIH faculty and clinical partners also suggest that teams were more effective after team training than before. Future studies could be strengthened by having a concurrent “control” class or cohort as well as by formally including clinical partners and/or faculty in evaluation of individual or team outcomes (i.e., whether they felt the team worked better or whether their experience was more positive). Another challenge that we faced was the difficulty in collecting meaningful baseline data for teams as they were forming. As a result, we collected data at two time points – the end of the first quarter and the end of the last quarter of the program and compared data both between and within years and across years. Due to concerns about survey burden, we only collected data at two time points rather than three (e.g., at the beginning of Q1). Survey-based self-report research also presents challenges (including issues of ceiling effects), and in future studies we hope to utilize more mixed methods approaches [40,41]. As a first study of team science integration within engineering education, we have found survey results to be informative in our own evaluation of the effectiveness of team science content implementation. We anticipate that the results of our study to integrate team science training into an existing engineering course will be of value to other engineering programs. We also anticipate that this model will be relevant to clinical and translational research teams outside of the classroom setting. Future studies would also benefit from tracking outcomes over a longer time horizon to determine if future grants, manuscripts, patents, or collaborations resulted.

We collaboratively tailored, implemented, and evaluated team science training content and approaches in an existing year-long longitudinal engineering program tackling clinical and translational health challenges at the UW. Team training during the first year of implementation was well received by students and faculty. Post-implementation surveys of students demonstrate measurable improvement in individual self-efficacy and interpersonal team climate.

In this paper, we describe an innovative model for integrating team science training within an existing education program – both in terms of approach and audience – and present preliminary evidence of effectiveness. The collaborative train-the-trainer approach to adapting, implementing, and evaluating our work has led to a sustainable model within the EIH program. During the third year of this effort, EIH faculty led all of the team science sessions with asynchronous support from the Team Science Core. This work suggests that this approach is likely sustainable at the UW and replicable and adaptable to other clinical and translational research settings and audiences. We tailored existing content from other team trainings that we have carried out for research, education, and clinical teams to this project [18,22,42–44].

While there has been a growing acknowledgement of the need for team science training for interdisciplinary translational research teams, we are not aware of any previous studies involving student-professional teams [45]. In the engineering literature, multiple programs have incorporated a collaborative student-professional design approach to address unmet health needs. Ours may be the first to intentionally integrate team science training for student-professional teams and to track student team outcomes. Our work extends both the team science and engineering education literature by providing a model for the implementation and evaluation of intentional integration of team science training for interdisciplinary student-professional teams. It also adds to the nascent body of literature describing team science training models for clinical and translational teams.

A lack of best practices exists as to how to introduce and facilitate development of effective functioning among teams working together with a common goal (e.g., the EIH project). Replicable approaches are needed to enhance teamwork competence among interdisciplinary teams carrying out clinical and translational work. Here, we describe the collaborative adaption, implementation, and evaluation of team science content and approaches in an existing year-long longitudinal engineering program focused on unmet clinical and translational health challenges. Team science training provided to engineering student-professional teams resulted in measurable improvements in self-efficacy to participate in interdisciplinary teams and in interpersonal team climate (i.e., psychological safety) within those teams.

## Supplementary material

For supplementary material accompanying this paper visit https://doi.org/10.1017/cts.2021.788. click here to view supplementary material
