# Supplementary material for: Implementation and evaluation of team science training for interdisciplinary teams in an engineering design program
Source: J Clin Transl Sci. 2021 May 14;5(1):e127. doi: 10.1017/cts.2021.788 (PMC8327544; doi:10.1017/cts.2021.788)
Supplement: Supplementary file 1 [file S2059866121007883sup.zip › S2059866121007883sup001.docx]

**Supplementary Material**

**Appendix A. Surveys administered in EIH during Q1 and Q3**

Two surveys were administered in the EIH class each year. The Q1 surveys were administered at the end of Q1 and asked students to respond to questions related to working on teams before and after working in their EIH project teams. The Q3 surveys were administered at the end of Q3 and asked students to respond to questions related to their team experience specifically with their EIH project team. The questions are provided below for the Q1 survey and the Q3 survey.

Q1 Survey Questions

**Part 1: Please think about the times you have worked on group projects or teams in different settings in the past.**

1. Prior to your participation in this class, what types of settings have you worked on group projects or teams? (select all that apply)
2. High School Classes
3. College (outside of engineering classes)
4. College (in engineering classes)
5. Work (paid or volunteer)
6. None
7. Based on your *past* *experience working on group projects or teams* in all the settings you selected in question 1 above (prior to enrolling in EIH), please rate your capability of each statement. [*Matrix of options:* *not at all capable (1), somewhat capable, neither capable nor incapable, somewhat capable, very capable (5)*]
   1. Speak up in team meetings
   2. Effectively contribute in team meetings
   3. Recognize team members’ strengths
   4. Resolve conflicts with peers and other project collaborators
   5. Advocate for multiple points of view (different perspectives)
   6. Have your voice heard in team meetings
   7. Collaborate with team members who have different working styles
   8. Clarify language differences across disciplines/backgrounds (e.g., differences in terminology)

**Part 2: Please answer the following questions about your experience so far of working this term with your EIH project team.**

1. Please rate how satisfied you were in working with your EIH project team this quarter. [*Very dissatisfied (1), dissatisfied, neutral, satisfied, very satisfied (5)*]
   1. Please explain your response.
2. Please rate how satisfied you are with the progress made on your EIH project this quarter. [*Very dissatisfied (1), dissatisfied, neutral, satisfied, very satisfied (5)*]
   1. Please explain your response.
3. Based on your *current experience working in your EIH project team*, please rate your capability of each statement. [*Matrix of options:* *not at all capable (1), somewhat capable, neither capable nor incapable, somewhat capable, very capable (5)*]
4. Speak up in team meetings
5. Effectively contribute in team meetings
6. Recognize team members’ strengths
7. Resolve conflicts with peers and other project collaborators
8. Advocate for multiple points of view (different perspectives)
9. Have your voice heard in team meetings
10. Collaborate with team members who have different working styles
11. Clarify language differences across disciplines/backgrounds (e.g., differences in terminology)
12. Based on your current experience working in your EIH project team, please rate your agreement with each statement. [*Matrix of options:* *strongly disagree (1), disagree, neutral, agree, strongly agree (5)*]
13. Our project team has been successful working together
14. Our project team has a climate of collaboration and trust
15. I felt comfortable giving my team members feedback
16. I felt comfortable receiving feedback from my team members
17. Team members on my project had a high level of mutual trust in each other
18. I had a desire to know my team members on a personal level
19. Having a successful project was a priority for me
20. Building effective relationships with my team members was a priority for me
21. I was comfortable showing the limits or gaps in my knowledge with my team members
22. Communication with my team members outside of class (e.g., scheduling team meetings) was easy
23. Please briefly describe a “best practice” you found to facilitate successful teamwork with your EIH project team.
24. What other ideas do you have or have you seen that would facilitate successful teamwork for EIH project teams?
25. Please briefly describe one challenge you have encountered in working with your EIH project team and how your team did or did not overcome the problem.
26. How would you describe your main role(s) in your EIH project team? How were these roles determined? Did your role(s) change over time?

**Part 3: Please answer the following questions about your experience of working this term with your EIH faculty and clinical partners.**

1. Based on your experience this quarter with EIH faculty, please rate your agreement each statement. [*Matrix of options:* *strongly disagree (1), disagree, neutral, agree, strongly agree (5)*]
2. Communication with EIH faculty outside of class (e.g., to get advice or ask questions) was easy
3. I felt comfortable sharing team project updates with EIH faculty
4. I felt comfortable receiving feedback from EIH faculty
5. Building effective relationships with EIH faculty was a priority for me
6. I was comfortable showing the limits or gaps in my knowledge with EIH faculty
7. Based on your experience this quarter with your EIH clinical partner(s), please rate your agreement each statement. [*Matrix of options:* *strongly disagree (1), disagree, neutral, agree, strongly agree (5)*]
8. Communication with EIH clinical partners outside of class (e.g., to get advice or ask questions) was easy
9. I felt comfortable sharing team project updates with EIH clinical partners
10. I felt comfortable receiving feedback from EIH clinical partners
11. Building effective relationships with EIH clinical partners was a priority for me
12. I was comfortable showing the limits or gaps in my knowledge with EIH clinical partners
13. ^[[1]](#footnote-1)^Of the following activities that were introduced by the Team Science faculty, please indicate the extent to which each was useful to improve the effectiveness of your EIH project. [*Matrix of options:* *not at all helpful (1), slightly helpful, moderately helpful, helpful, very helpful (5)*]
    1. Impromptu networking (when you were asked to rotate partners 3 times with a question prompt)
    2. Developing a Team Agreement
    3. Writing a Welcome Letter as a team
    4. Having a structured approach during the initial in-class meeting with clinical partners
    5. In-class session on giving and receiving feedback in preparation for mid-term presentations
    6. Having a structured approach to giving and receiving feedback during the mid-term presentations
    7. Interviewing and being interviewed during the appreciative inquiry session
14. Is there anything else about your experience in this EIH class or working in your project team that you would like to share? Note: If you have a comment that needs a personalized response, please reach out to the instructor.

**Part 4: Please tell us a little about yourself so that we can examine whether there are differences in experience for students according to their demographic and/or educational background:**

1. Is English your first language? [*Yes, No*]
2. What is your gender? [*Male, Female, prefer not to say]*
3. In what type of educational program are you enrolled? [*Undergraduate, graduate, other*]

Q3 Survey Questions

**Part 1: Please answer the following questions about your experience of working the past two terms with your EIH project team.**

1. Please rate how satisfied you were in how your team worked together to complete your EIH project during Q2 and Q3. [*very dissatisfied, dissatisfied, neutral, satisfied, very satisfied*]
   1. Please explain your response.
2. Please compare how satisfied you are in working with your EIH project team during Q2 and Q3 *as compared to Q1*. [*Much more satisfied with Q2/Q3, slightly more satisfied with Q2/Q3, neutral, slightly more satisfied with Q1, much more satisfied with Q1*]
   1. Please explain your response.
3. Please rate how satisfied you are with your current EIH product. [*very dissatisfied, dissatisfied, neutral, satisfied, very satisfied*]
4. Based on your current experience working with your current EIH project team, please rate your capability of each statement. [*Matrix of options:* *not at all capable (1), somewhat capable, neither capable nor incapable, somewhat capable, very capable (5)*]
   1. Speak up in team meetings
   2. Effectively contribute in team meetings
   3. Recognize team members’ strengths
   4. Resolve conflicts with peers and other project collaborators
   5. Advocate for multiple points of view (different perspectives)
   6. Have your voice heard in team meetings
   7. Collaborate with team members who have different working styles
   8. Clarify language differences across disciplines/backgrounds (e.g., differences in terminology)
5. Based on your current experience working with your EIH project team, please rate your agreement each statement. [*Matrix of options:* *strongly disagree (1), disagree, neutral, agree, strongly agree (5)*]
6. Our project team has been successful in working together
7. Our project team has a climate of collaboration and trust
8. I felt comfortable giving my team members feedback
9. I felt comfortable receiving feedback from my team members
10. Team members on my project had a high level of mutual trust in each other
11. I had a desire to know my team members on a personal level
12. Having a successful project was a priority for me
13. Building effective relationships with my team members was a priority for me
14. I was comfortable showing the limits or gaps in my knowledge with my team members
15. Communication with my team members outside of class (e.g., scheduling team meetings) was easy
16. Please briefly describe a “best practice” you found to facilitate successful teamwork with your EIH project team. (open-ended response)
17. What other ideas do you have or have you seen that would facilitate successful teamwork for EIH project teams?
18. Please briefly describe one challenge you have encountered in working with your EIH project team and how your team did or did not overcome the problem.
19. How would you describe your main role(s) in your EIH project team? How were these roles determined? Did your role(s) change over time?
20. How likely is it that you and your EIH project team will participate in the following activities related to your EIH project. [*Matrix of options: highly unlikely (1), unlikely, neither likely nor unlikely, likely, highly likely, already in progress, completed (7)*]
21. Continue to work as a team after the official end of the EIH program year
22. Complete an IRB application to test your device on human subjects
23. Manufacture your device, to scale, using the materials you intend to use if your product were to be available for sale
24. Seek FDA approval for your device
25. Publish scholarly manuscripts related to your EIH project
26. Present EIH project device at university, local, regional, or national conferences or symposia
27. Complete patent application(s) related to your EIH project
28. Form a commercial entity
29. Apply for grants or seed funding to continue your work on the EIH project
30. Engage in other fundraising activities to continue your work on the EIH project
31. Receive grants, seed funding awards, or other sponsorship to continue your work on the EIH project
32. Obtain press or media coverage of your EIH device at the university, local, regional, or national level
33. Engage in other activities that demonstrate success or productivity on the EIH project
34. If other, please explain here.
35. What is your intention of staying in touch with your EIH project team members? [C*heck all that apply*]
    1. Personally
    2. On this particular project
    3. In school
    4. Professionally
    5. No intention of staying in touch

**Part 2: Please answer the following questions about your experience of working with EIH faculty, clinical partners, Team Science, and guest experts.**

1. Based on your experience this year with your EIH faculty, please rate your agreement each statement. [*Matrix of options:* *strongly disagree (1), disagree, neutral, agree, strongly agree (5)*]
2. Communication with EIH faculty during class sessions (e.g., to get advice or ask questions) was easy
3. I felt comfortable sharing team project updates with EIH faculty
4. I felt comfortable receiving feedback from EIH faculty
5. Building effective relationships with EIH faculty was a priority for me
6. I was comfortable showing the limits or gaps in my knowledge with EIH faculty
7. Communication with EIH faculty outside of class (e.g., to get advice or ask questions) was easy
8. Please describe the role of your EIH faculty and what it was like working with them. How did EIH faculty promote or inhibit effective teamwork and/or productivity?
9. Based on your experience this year with your EIH clinical partner(s), please rate your agreement each statement. [*Matrix of options:* *strongly disagree (1), disagree, neutral, agree, strongly agree (5)*]
10. I felt comfortable sharing team project updates with EIH clinical partners
11. I felt comfortable receiving feedback from EIH clinical partners
12. Building effective relationships with EIH clinical partners was a priority for me
13. I was comfortable showing the limits or gaps in my knowledge with EIH clinical partners
14. Communication with EIH clinical partners outside of class (e.g., to get advice or ask questions) was easy
15. Please describe the role of your clinical partners and what it was like working with them. How did clinical partners promote or inhibit effective teamwork and/or productivity?
16. Of the following activities that were introduced by the Team Science faculty, please indicate the extent to which each was useful to improve the effectiveness of your EIH project. [*Matrix of options: not at all helpful (1), slightly helpful, moderately helpful, helpful, very helpful (5)*]
17. Shift-and-share presentations
18. Personal and social styles
19. TROIKA consulting
20. Thomas-Kilmann Conflict Mode Instrument (TKI)
21. Worksheet for planning difficult conversations
22. Response to conflicting feedback – evaluation of options
23. Please indicate the extent to which the team science sessions/tools helped your team to become more [*Matrix of options: not at all helpful (1), slightly helpful, moderately helpful, helpful, very helpful (5)*]
    1. Efficient (well-organized; minimum wasted effort; reaching milestones in a timely manner; etc.)
    2. Effective (successful in producing desired or intended result; completing milestones; etc.)
    3. Successful in carrying out your project together

**Part 3: Please tell us a little about yourself so that we can examine whether there are differences in experience for students according to their demographic and/or educational background:**

1. Is English your first language? [*Yes, No*]
2. What is your gender? [*Male, Female, prefer not to say]*
3. In what type of educational program are you enrolled? [*Undergraduate, graduate, other*]
4. Is there anything else about your experience in this EIH class or working with your EIH project team, EIH faculty, or clinical partners that you would like to share? Note: If you have a comment that needs a personalized response, please reach out to your course instructors.

**Appendix B. Team science session and tool details**

| **Session** | **Timing** | **Objectives** | **Tools Used** |
| --- | --- | --- | --- |
| Team dynamics 101 | Q1 | - Provide team science background and rationale - Share prior team experiences to open discussion around effective team functioning - Begin to create relationships with classmates | Impromptu Networking, 1-2-4-All, PollEV |
| Team formation (1) | Fall  Week 1 | - Understand team structures and functions - Establish relationships with team members - Develop team agreements - Learn and practice meeting management skills | TRIZ, PollEV, Meeting Roles, Team Agreements, Welcome Letter |
| Team formation (2) | Fall  Week 2 | - Draft a vision statement for the project - Identify stakeholders and draft needs statements - Practice meeting roles and management skills | Flow Mapping, PollEV |
| Giving and receiving feedback | Fall  Week 4 | - Gain knowledge and skills to give and receive constructive feedback - Enable students to continue to build positive, confirming relationships | Tips for providing written peer review |
| Team dynamics reflection | Fall  Week 9 | - Reflect on multidisciplinary team dynamics - Reflect on and practice articulating specific contributions to the team, as well as recognizing the contributions of other team members | Appreciative Inquiry |
| Team regroup | Winter  Week 1 | - Facilitate incorporation of new team members - Provide tools to help teams identify and meet goals | AOIS |
| Design review | Winter  Week 3 | - Quickly gather feedback from classmates and instructors about initial prototypes - Practice and refine project pitch - Discover innovative approaches used by other teams | Shift & Share |
| Personal styles | Winter  Week 8 | - Develop basic understanding of personal styles - Identify styles of teammates and self - Develop understanding of versatility and explore strategic uses of styles to promote team success | PSI |
| Design review | Winter  Week 10 | - Quickly gather feedback - Practice and refine project pitch - Discover innovative approaches | Shift & Share |
| Peer consulting | Spring  Week 2 | - Practice identifying and articulating challenges or assistance needed - Practice providing constructive feedback | TROIKA |
| Constructively engaging in conflict | Spring  Week 3 | - Identify purpose and methods of constructively engaging with conflict - Develop understanding of conflict resolution mode preferences - Learn and practice tools to prepare for and engage in conflict resolution | TKI, Difficult Conversation Worksheet |
| Design review | Spring  Week 5 | - Quickly gather feedback - Practice and refine project pitch - Discover innovative approaches | Shift & Share |
| Navigating feedback | Spring  Week 7 | - Expand understanding of how to navigate and incorporate feedback - Learn tools & practice strategies for making project decisions | FFA, Judgement Map |

# **Team Science Tool Reference Guide**

Impromptu Networking. This Liberating Structure (LS) activity allows individuals in a new group to build loose yet powerful connections within a matter of minutes by asking engaging questions. Briefly, pairs discuss a prompt (90 seconds per individual) and repeat 2 more times with a new partner. <http://www.liberatingstructures.com/2-impromptu-networking/>

**1-2-4-All.** This LS exercise is useful for engaging everyone simultaneously in generating questions, ideas, and suggestions. This activity starts with silent self-reflection (1 min), generation of ideas in pairs (2 min), sharing and development of ideas in foursomes (4 min), and reporting out to whole group (5 min). Implementation of this activity in EIH was often paired with the PollEV tool to allow students to project responses in front of the classroom should the reporting out to the whole group not get around to everyone. <http://www.liberatingstructures.com/1-1-2-4-all/>

**TRIZ.** This LS activity clears space for innovation by helping a group let go of what it knows (but rarely admits) limits its success and inviting creative destruction. Structured in a 3-step process, groups first brainstorm a list of what makes for an undesirable outcome, then identify ways in which the individual or team partakes in items on the list, and finally strategize how to stop and prevent this type of behavior that creates undesirable results. <http://www.liberatingstructures.com/6-making-space-with-triz/>

**Shift-and-Share.** This LS activity transforms long, large-group presentations into several, concise small-group discussions. Design reviews in the EIH program were structured in a shift-and-share format to facilitate interactive feedback and allow participants opportunity to learn from repetition of the presentation. The design review sessions were split into 2 rounds, during which half of the teams were presenters and half of the teams were audience members. The audience split into small groups and rotated through the presenting team stations. Each presenting team gave a concise description of their innovations (4-5 minutes), then for the next 10 minutes the audience asked questions and provided feedback. <http://www.liberatingstructures.com/11-shift-share/>

**Appreciative Interviews.** This LS activity is a positive, collaborative, strengths-based approach to leadership development & innovative organizational change. Appreciative interviews were used towards the end of the first quarter of the course to invite student reflection on the role of team dynamics in their project teams in preparation for the transition to the second quarter of the course. A worksheet was provided with prompt questions and to provide space for note-taking. Participants created pairs and took turns interviewing each other for 10 minutes each. An 8-minute small group debrief was then conducted, followed by a 5-minute large group debrief. <http://www.liberatingstructures.com/5-appreciative-interviews-ai/>

**TROIKA Consulting.** This LS activity is designed to create space for peer-to-peer advice. In this activity, a prompt is proposed such as “what is a challenge you are facing?” or “what do you need help with?” Students split into groups of 3, with each trio having members from different groups. During each round, one member of the trio serves as the “client” and the other 2 members serve as the “consultants.” The client reflects on and discusses the prompt to the consultants first, then the consultants discuss the client’s response to the prompt with each other for the next XX minutes. During the “consulting period” the client has their back turned to the consultants, to invite full listening. This activity is repeated 2 more times, with a new member of the group serving as the client each round. <http://www.liberatingstructures.com/8-troika-consulting/>

**Team Agreements.** Team agreements are a set of expectations regarding team functioning that serve to prevent conflict by creating a shared understanding of expectations, establishing team culture, and providing an objective point of reference for feedback or discussion should conflict arise.

**Team Welcome Letter.** Team welcome letters were created by project teams in the EIH program to facilitate development of a shared project vision within the team. The letters included an introduction that specified the clinical need, the team’s goals, team agreements, communication plan, and a contingency plan for addressing potential challenges/conflict.

**Poll Everywhere (PollEV).** PollEV is a web-based audience response system that allows speakers to embed interactive activities (e.g., s polls, open-ended questions, etc.) directly into presentations, to which the audience responds on the web or via SMS texting on their phones. <https://pollev.com/>

**CATME.** Comprehensive Assessment of Team Member Effectiveness (CATME) is a fee-based set of tools designed to help instructors manage group work and team assignments more effectively, and has been used in the EIH program to assess peer evaluations. <https://info.catme.org/>

**Personal and social styles.** The personal and social styles activity is used to increase awareness of interpersonal functioning identifying an individual’s dominate personality style. Each of the 4 styles (driver, expressive, amiable, analytical) describes a person’s preferred communication approaches and behaviors as perceived by others along 2 dimensions: assertiveness and responsiveness. This tool was used in the course to facilitate identification of personal styles within teams and provide background knowledge and tools to enhance effectiveness in working with other styles.

**Thomas-Kilmann Instrument (TKI).** The TKI is a self-assessment instrument designed to measure a person’s behavior in conflict situations along 2 dimensions: “(1) assertiveness, the extent to which the person attempts to satisfy their own concerns, and (2) cooperativeness, the extent to which the person attempts to satisfy the other person’s concerns.” Students completed TKI assessments and conflict modes were discussed during class to provide insight into effective conflict resolution with knowledge of the conflict modes. <https://kilmanndiagnostics.com/overview-thomas-kilmann-conflict-mode-instrument-tki/>

**Difficult Conversations Worksheet.** This tool is used to prepare for a difficult conversation by encouraging internal reflection on the issue at hand and the perspectives of the parties involved.

**Meeting Roles.** Team members were encouraged to use the following roles: leader, facilitator, timekeeper, recorder, and participant.

**Name Story.** The Name Story is an activity designed to help students remember the names of their classmates through personal connections and to realize the variety of cultures and histories that are brought to the classroom. In this activity, students share their name and any history or story associated with their name such as the meaning of the name or why they were given or chose that name. <https://sites.lsa.umich.edu/inclusive-teaching/2017/08/23/name-story/>

**Debrief.** Debriefs were conducted at the end of each team science training session for continued training content development. In each debrief, the Team Science instructors prompted the audience to discuss what went well and what they thought could be improved.

**Judgement Model.** A judgement model is a tool used to evaluate and compare 3 to 5 mutually exclusive options. Users of the tool list the options along the left side of a matrix, and list criteria used in making the selection of an option along the top of the matrix. Each option is then ranked against each criterion on a scale of 1 (low) to 5 (high). The criteria scores are summed for each option to indicate the strongest solution based on the evaluated criteria.

**Force Field Analysis (FFA).** FFA was developed in the 1940s by Kurt Lewin, and provides a structured decision-making framework. To conduct an FFA, the user of the tool specifies a proposed solution, modification, plan, etc. The user then identifies driving forces that encourage the proposed change, as well as the restraining forces that are obstacles to the proposed change. The strength of each force is then rated from 1 (weak) to 5 (strong) and a total score for each side is calculated. This analysis can be used to determine if successful change is likely, or if action must be taken on certain forces to modify their strength.

**Flow Mapping.** This planning and management tool is used to visually describe the flow of work, goods, users, or other items. In the EIH course, flow mapping of clinical stakeholders was used to identify the people involved with or affected by the problem, the people who interact with clinical stakeholders, and the connections between these people. Additionally, flow mapping of the design team and resources available to the team was used to facilitate recognition of the people, departments, funding opportunities, and physical resources to be leveraged by each team.

**AOIS.** The AOIS tool is a template document that prompts teams to identify and discuss their current accomplishments, objectives, issues, and schedule. EIH teams completed this project management tool and reviewed their AOIS with faculty each week during the winter and spring quarters.

**Appendix C. Expected project outcomes**

In the Q3 surveys, students were asked to respond to questions related to project outcomes. A visual comparison of the responses to statements between Y0 (lower rows) and Y1 (upper rows) is shown in **Appendix Figure 1**.

**Appendix Figure 1.** A visual comparison of responses to statements related to expected project outcomes between Y0 (lower rows) and Y1 (upper rows).

*Note:* Responses are based on students reporting their level of likeliness on a 5-point Likert-type scale (red, grey, and green shades), or if progress or completion has already been established (blue shades).

1. Note that question 13 was only included in the Y1 survey when team science training was implemented in the program. [↑](#footnote-ref-1)
